# DNA-Protein Vaccination Strategy Does Not Protect from Challenge with African Swine Fever Virus Armenia 2007 Strain

**DOI:** 10.3390/vaccines7010012

**Published:** 2019-01-28

**Authors:** Sun-Young Sunwoo, Daniel Pérez-Núñez, Igor Morozov, Elena G. Sánchez, Natasha N. Gaudreault, Jessie D. Trujillo, Lina Mur, Marisa Nogal, Daniel Madden, Kinga Urbaniak, In Joong Kim, Wenjun Ma, Yolanda Revilla, Juergen A. Richt

**Affiliations:** 1 Department of Diagnostic Medicine & Pathobiology, College of Veterinary Medicine, Kansas State University, K2224 Mosier Hall, 1800 Denison Ave, Manhattan, KS 66506, USA; sunwoosy@gmail.com (S.-Y.S.); imorozov@vet.k-state.edu (I.M.); nng5757@vet.k-state.edu (N.N.G.); jdtrujillo@vet.k-state.edu (J.D.T.); linamurvet@gmail.com (L.M.); dwmadden@vet.k-state.edu (D.M.); kinia-u@wp.pl (K.U.); lui0125@gmail.com (I.J.K.); wma@vet.k-state.edu (W.M.); 2 CBMSO-CSIC-UAM, C/Nicolás Cabrera 1, Campus de Cantoblanco, 28049 Madrid, Spain; daniel_perez@cbm.csic.es (D.P.-N.); elena_garcia@cbm.csic.es (E.G.S.); marisa_nogal@cbm.csic.es (M.N.)

**Keywords:** African swine fever virus, subunit vaccine, plasmid-expressed antigen, recombinant protein, immune response, immunopathology, Armenia 2007 strain

## Abstract

African swine fever virus (ASFV) causes high morbidity and mortality in swine (*Sus scrofa*), for which there is no commercially available vaccine. Recent outbreaks of the virus in Trans-Caucasus countries, Eastern Europe, Belgium and China highlight the urgent need to develop effective vaccines against ASFV. Previously, we evaluated the immunogenicity of a vaccination strategy designed to test various combinations of ASFV antigens encoded by DNA plasmids and recombinant proteins with the aim to activate both humoral and cellular immunity. Based on our previous results, the objective of this study was to test the combined DNA-protein vaccine strategy using a cocktail of the most immunogenic antigens against virulent ASFV challenge. Pigs were vaccinated three times with a cocktail that included ASFV plasmid DNA (CD2v, p72, p32, +/−p17) and recombinant proteins (p15, p35, p54, +/−p17). Three weeks after the third immunization, all pigs were challenged with the virulent ASFV Armenia 2007 strain. The results showed that vaccinated pigs were not protected from ASFV infection or disease. Compared to the non-vaccinated controls, earlier onset of clinical signs, viremia, and death were observed for the vaccinated animals following virulent ASFV challenge. ASFV induced pathology was also enhanced in the vaccinated pigs. Furthermore, while the vaccinated pigs developed antigen-specific antibodies, immunized pig sera at the time of challenge lacked the capacity to neutralize virus, and instead was observed to enhance ASFV infection in vitro. The results of this work points to a putative immune enhancement mechanism involved in ASFV pathogenesis that warrants further investigation. This pilot study provides insight for the selection of appropriate combinations of ASFV antigens for the development of a rationally-designed, safe, and efficacious vaccine for ASF.

## 1. Introduction

African swine fever (ASF), is caused by a large enveloped DNA virus (ASFV) of the family *Asfarviridae* [1,2]. Virulent ASFV strains cause acute hemorrhagic fever in domestic pigs and wild boar (*Sus scrofa*) with up to 100% mortality within 12 days after infection, while other ASFV strains cause subacute or mild disease [3]. ASF is considered one of the most significant and devastating viral diseases of domestic pigs primarily due to the lack of effective vaccines [4]. To date, vaccination strategies have been proposed with a range of technologies including live-attenuated and inactivated viruses, recombinant proteins/peptides, among others [5,6]. Unfortunately, the development of an effective, safe ASFV vaccine has been largely unsuccessful due to the complexity of the virus. ASFV encodes for more than 150 viral proteins, making the identification of individual, or combinations of, viral antigens that may elicit a protective response against ASF challenging [7]. Several ASFV immunodominant proteins have been identified [8]. However, the determinants for protection against ASF is still not fully understood, but appears to rely on multiple immune mechanisms which adds another layer of complexity [9].

Attenuated or low virulent ASFV strains have been shown to induce protective immune responses against homologous or rarely heterologous virulent ASFV strains [10,11,12,13]. Unfortunately, these viruses can produce adverse side effects, such as persistent chronic infections that can lead to chronic lesions of the skin and joints, lymphadenopathy, pneumonia, recurrent fever, chronic viremia, and hypergammaglobulemia [10,11,12]. Furthermore, their application in non-ASFV-endemic regions raises important concerns of safety and trade restrictions. Therefore, subunit- and vector-based vaccine strategies have been investigated for their safety and ability to elicit virus neutralizing antibodies and enhance cellular immune responses. 

ASFV-specific antibodies have been shown to protect pigs against a lethal homologous ASFV challenge, demonstrating the protective properties of ASFV-specific antibodies [14,15,16]. Yet, the specific role and relative importance of neutralizing antibodies in protection is not clear [17]. ASFV proteins reported to induce neutralizing antibodies in immunized pigs include p72, p54, and p32 (also called p30) [18,19]. Pigs immunized with either p54 or p32 recombinant proteins alone were not protected against lethal infection, although immunization with p54 and p30 combined did delay disease and offer some protection against challenge [20]. Immunization with baculovirus-expressed ASFV hemagglutinin (HA) protein, CD2v encoded by EP402R, also showed some degree of protection against virulent virus challenge in the presence of specific antibodies [21]. However in another study, pigs immunized with a cocktail of baculovirus-expressed ASFV proteins (p32, p54, p72, and p22) produced neutralizing antibodies, but were not protected from virulent challenge [22]. 

Growing evidence indicates the cytotoxic T-lymphocyte (CTL) mediated cellular immune response also plays a critical role in clearing ASFV infection [23,24]. For example, DNA immunization with the extracellular domain of CD2v fused to p32 and p54 failed to provide protection against virus challenge; however, fusion of these ASFV-determinants to ubiquitin, conferred partial protection from lethal challenge in the absence of specific antibodies, which correlated with the proliferation of antigen specific CD8+ T-cells [25]. A baculovirus BacMam-expressed CD2v/p32/p54 fused antigen also provided partial protection [26]. Furthermore, immunization with an ASFV DNA expression library that lacked CD2v, p32, and p54 conferred partial protection against virulent challenge [27]. Once again, protection correlated with the presence of virus specific T-cells and the absence of detectable antibodies, highlighting the role of the CTL-mediated immune response in protection. In addition, it revealed the existence of multiple additional ASFV antigens with potential protective capacity. Together these studies suggest that DNA and protein-based vaccines induce a specific antiviral immune response based on either T-cells or neutralizing antibodies, that can provide at least partial protection against ASFV challenge. 

Based on these and other studies of ASFV proteins [20,28,29,30,31,32,33], we designed an immunogenicity study to test various combinations of ASFV-specific plasmid-expressing copy DNAs (pcDNAs) and recombinant proteins on the ability to elicit both cellular and humoral immune responses. Previously, we evaluated antigen-specific immune responses to various combinations of single pcDNA and proteins, as well as various mixtures of each, in pigs [34]. The results of that study led us to test a mixture of combined ASFV proteins and pcDNAs in a pilot vaccination-challenge study. In this pilot study, viral antigens shown previously to induce cellular immune responses (p15 and p72) and humoral immune responses (CD2v, p72, p54, p32, and p35), were combined in a DNA-protein vaccine cocktail. In addition, the ASFV inner envelope protein, p17, which we hypothesize may improve immunogenicity was also included in the vaccine cocktail for one of the groups. The vaccination approach included two boosts at days 21 and 35, followed by virulent challenge 3 weeks after the last immunization. Immunogenicity of our vaccine strategy was accessed via determination of ASFV antigen-specific and neutralizing antibodies, and the development of antigen-specific CTL response by induction of interferon gamma (IFN-γ) secreting cells. Vaccine efficacy was evaluated against virulent challenge with the ASFV Armenia 2007 strain, and clinical, virological, and pathological endpoints quantitatively accessed.

## 2. Materials and Methods 

### 2.1. ASFV Recombinant Proteins and Plasmid DNAs for Immunization

A summary of the ASFV antigens used in this study and their source strains are shown in Table 1. A comparison of the percent sequence identities were obtained by performing nucleotide and protein BLAST analysis on NCBI (https://www.ncbi.nlm.nih.gov/). The recombinant ASFV proteins were prepared as previously described [34]. Briefly, full-length ORF of CP530R (coding for the p15 and p35) and E183L (coding for p54) were synthesized based on ASFV isolate Georgia/2007 sequence (GeneBank: FR682468.1) and cloned into a common plasmid vector (e.g., pUC57, GENEWIZ). ASFV proteins p15, p35, and p54 were expressed in baculoviruses using the BaculoDirect expression system (Invitrogen). In addition, the ASFV p17 protein (sequence based on the D117L ORF from Ba71V strain) was expressed using the pGEX 4T-1 *Escherichia coli* (*E. coli*) expression system (GE Healthcare Life Sciences, Chicago, IL, USA). Recombinant ASFV protein expression was confirmed by western blot using lysates from either baculovirus-infected SF9 cells or *E. coli* [34]. After confirmation of expression, recombinant proteins with histidine tags were purified via affinity chromatography using Ni-NTA superflow resin (Qiagen, Hilden, Germany) after lysis of cells under native condition. The p17 expressing *E. coli* pellet was lysed under denaturing condition and solubilized in 8M urea. All proteins were dialyzed against phosphate-buffered saline (PBS; pH 7.4, 150 mM NaCl, 4 mM EDTA, 10% glycerol) and aliquots were stored at −80 °C until use. 

The pcDNA 3.1 plasmids expressing ASFV-specific genes were prepared as described [34]. Briefly, overlapping PCR was used to insert the full-length genes EP402R (coding for CD2v), CP204L (coding for p32), B646L (coding for p72), and D117L (coding for p17) into pcDNA 3.1 (Invitrogen, Carlsbad, CA, USA) by adding restrictions sites to the amplification primers. The fragments were amplified by PCR using the specific primers for each gene from lysate of cells infected with the Ba71V strain (GenBank: NC_001659.2), with the exception of p72 which was amplified from cells infected with ASFV E70 (GenBank: AY578692.1). PCR products and pcDNA vector were digested with the corresponding restriction enzymes and ligated with T4 ligase (Roche). Products were used to transform *E. coli* DH5α by heat shock. Correctness of respective plasmid construct was confirmed by DNA sequencing and blast analysis.

### 2.2. Cells and Virus

Vero and COS-7 cells (both derived from African green monkey kidneys) were obtained from the American Type Culture Collection (ATCC) to use for virus propagation. Cells were cultured in Dulbecco’s Modified Eagle’s Medium (DMEM) supplemented with 2 mM L-glutamine, 100 U/mL gentamicin, nonessential amino acids, and 5% fetal bovine serum (FBS; Invitrogen Life Technologies), and maintained at 37 °C under a 7% CO_2_ atmosphere saturated with water vapor.

The Vero-adapted ASFV strain Ba71V and virulent strains E70 were propagated on Vero and COS-7 cells, respectively, as described previously [35]. In brief, sub-confluent monolayers were cultivated in cell culture treated roller bottles and infected with ASFV at a multiplicity of infection (MOI) of 0.5 in culture medium. At 72 h post infection, cells were pelleted and the supernatant recovered. Viral supernatant was centrifuged at 14,000 rpm for 6 h at 4 °C and the purified infectious virus was re-suspended in medium and stored at −80 °C. 

The Armenia 2007 (Arm07) isolate is classified as ASFV genotype II [36], similar to the Georgia 2007 strain, and was obtained from the European Union Reference Laboratory for ASF, Centro de Investigación en Sanidad Animal, Instituto Nacional de Tecnología Agraria y Alimentaria (CISA-INIA) and was used for virus challenge. Arm07 was propagated on primary alveolar macrophages as described previously [37]. 

### 2.3. Animals and Experimental Design

#### 2.3.1. Ethics Statement for Use of Animals

All animal studies and experiments were approved and performed under the Kansas State University (KSU) Institutional Biosafety Committee (IBC, Protocol #: 850) and the Institutional Animal Care and Use Committee (IACUC, Protocol #: 3513) in compliance with the Animal Welfare Act. The research related to ASFV was performed in biosafety level (BSL)-3 laboratory and facilities in the Biosecurity Research Institute (BRI) at KSU in Manhattan, KS, USA.

#### 2.3.2. Immunization of Animals

A total of ten, three-week old piglets were randomly divided into three groups. The piglets were acclimated for one week at the KSU Large Animal Research Center (LARC), a BSL-2 facility. Groups 1 (n = 3) and 2 (n = 2) animals were immunized with different combinations of recombinant proteins and pcDNA constructs, as shown in Table 2. Group 3 (n = 5) animals served as non-vaccinated controls. Piglets were inoculated intramuscularly (IM) with 100 μg of each respective recombinant protein mixed with ISA25 adjuvant (SEPPIC) and 100 μg of each pcDNA construct. Piglets were vaccinated three times at 0, 21, and 35 day post initial vaccination (dpv). Whole blood and serum samples were collected on the days of vaccination. 

#### 2.3.3. Virulent Challenge with ASFV Arm07 Strain

After the third immunization, the five vaccinated pigs and five non-vaccinated control pigs were transported from the LARC to the high containment BSL-3Ag facility at KSU BRI and acclimated for one week. Three weeks after the third immunization, the ten pigs were inoculated IM with 1 mL inoculum containing 360 HAU of Arm07. Whole blood and serum samples were collected daily from day 0 to 9 days post challenge (dpc). 

#### 2.3.4. Assessment of Clinical Signs

The clinical signs were evaluated and scored from 0 to 4 as shown in the Supplementary Table S1. Euthanasia via pentobarbital injection following sedation was performed if the accumulative clinical score was >16, i.e., when the pig had severe clinical signs or if warranted by the attending veterinarian. After challenge, rectal temperatures were measured once per day, then twice per day after temperature reached >41 °C.

### 2.4. Gross and Histological Pathology

Postmortem evaluation was performed on pigs euthanized or found dead following ASFV challenge. Gross and histological lesions were scored following standardized guidelines established by Galindo-Cardiel, et al. with minor modifications [38]. Briefly, gross evaluation was performed on epidermis, eyes, thoracic and abdominal cavities, primary and secondary lymphoid organs including the thymus and bone marrow, tonsils, and mandibular, cervical, tracheobronchial, gastrohepatic and renal lymph nodes. Additional tissues grossly evaluated included the thyroid gland, trachea, lung, heart, oral cavity, esophagus, stomach, small and large intestine, pancreas, liver and gall bladder, kidney, urinary bladder, and bone marrow. Gross lesion characterization and classification per organ was performed blindly by one pathologist for body condition, lungs, liver, spleen, kidney, and lymph nodes. Lesion classification was established as absent (0) or present at mild (1), moderate (2), or severe (3) with the numerical score established for each organ. Specific scoring schemes for each organ are described in Supplementary Table S2.

Standardized histological evaluation was performed blindly and independently by two pathologists on lung, liver, spleen, kidney, tonsil, and lymph nodes including submandibular, superficial cervical, perihepatic, mesenteric, and renal, as described by Galindo-Cardiel et al. with minor modifications [38]. Additional tissues evaluated histologically included heart, adrenal glands, skin and gastrointestinal tract. Lesion classification was established as absent (0) or present at mild (1), moderate (2), or severe (3) with the numerical score established for each organ. Specific scoring schemes for each organ are described in Supplementary Table S2. 

### 2.5. Detection of Serum Antibody by ELISA

Enzyme-linked immunosorbent assays (ELISAs) were performed using each of the recombinant ASFV proteins used for immunization as antigens. Diluent, washing solution and stop solution were used from the INGEZIM PPA DAS kit (Ingenasa, Madrid, Spain). Briefly, wells were coated with 200 ng of the respective recombinant ASFV protein in 100 µL of PBS and incubated overnight at 4 °C. Sera collected from immunized pigs at 49 dpv were incubated at dilution 1/20 and 1/200 in 100 µL of diluent at 37 °C for 1 h and washed four times with washing solution. Anti-pig IgGs conjugated with peroxidase (kindly gifted by Dr. E. Tabarés, UAM, Madrid, Spain) [32], diluted 1/5000 in 100 µL, was incubated at room temperature (RT) for 1 h protected from light. The colorimetric substrate, TMB, was added and incubated at RT protected from light. After 15 min, 100 µL of stop solution (2N sulfuric acid) was added. The OD (optical density) value was measured at 405 nm within 5 min of adding the stop solution. The assay was performed as biological triplicates and duplicates, for Groups 1 and 2 sera, respectively.

### 2.6. Detection of IFN-γ Secreting Cells (ELISPOT)

Blood from immunized pigs was collected into sodium heparin containing vacutainer tubes. Peripheral Blood Mononuclear Cells (PBMCs) were isolated by 1.077 Ficol-hypaque separation and suspended in complete RPMI-1640 media (Advanced RPMI 1640 medium; Thermo Fisher Scientific, Waltham, MA, USA,) supplemented with 10% FBS (Corning™, New York, USA) and antibiotic-antimycotic solution (Corning™). 

Antigen-specific IFN-γ response was determined by an enzyme-linked immunospot (ELISPOT) assay using anti-pig IFN-γ antibodies (P2G10 RUO or biotinylated anti-IFN-γ P2C11 RUO, Becton Dickinson Pharmingen) as per manufacturer’s instructions [39,40]. MultiScreenHTS IP 96 well filter plates (Millipore) were coated with 5 μg/mL of anti-IFN-γ antibody (P2G10RUO, BD Pharmingen, San Jose, CA, USA) by incubating at 4 °C overnight; afterwards plates were washed five times with sterile PBS. The well was blocked using medium with 10% FBS for 2 h at 37 °C. PBMCs were added to wells at a density of 2 × 10^5^ cells/well after decanting the blocking medium. Affinity-purified ASFV antigens were added to the wells at a final concentration of 6.0 μg/mL for proteins and 1.0 μg/mL for pcDNA plasmids, all done in duplicates. Concanavalin A (Con A) mitogen (6 μg/mL) was used as a positive control, and the pcDNAs and media alone mock were included as negative controls. 

Following 36 h incubation at 37 °C with 5% CO_2_, plates were washed with PBS containing 0.05% Tween 20 and 0.5 μg/mL of biotinylated anti-IFN-γ antibody (P2C11 RUO, BD Pharmingen) was added for 2 h at RT. Afterwards plates were washed with PBS containing 0.05% Tween 20, and Streptavidin-HRP (BioLegend, San Diego, CA, USA) in PBS with 0.5% FBS was added (1:2000 dilution). After an incubation period of 45 min at RT, the plates were washed with PBS and colorimetric staining was performed with the NovaRED solution (Vector labs, Burlingame, CA, USA) according to manufacturer’s instructions; then the plates were allowed to dry overnight. The spots were counted by a CTL ImmunoSpot Analyzer using ImmunoSpot 5.0.3 software (Cellular Technology Limited (CTL), Shaker Heights, OH, USA). 

### 2.7. Virus Neutralization Assay

Sera from pigs collected before vaccination (pre-immune sera) and at 49 dpv (immune sera) were incubated at 56 °C for 30 min for inactivation and diluted 1/8, using DMEM supplemented with 20% of FBS and 0.05% Tween-80. The level of virus neutralization was determined by identifying the percentage of ASFV-infected cells using fluorescence activating cell sorting (FACS) as described previously [34,41,42]. Briefly, sera samples diluted 1/8, previously incubated overnight with 1 × 10^6^ pfu of ASFV-Ba71V virus, were used to infect Vero cells for 16 h; the percentage of infected cells was then determined by detection of the major viral capsid protein p72. Cells were detached with trypsin-EDTA, fixed with 2% paraformaldehyde for 30 min at 4 °C and then permeabilized with PBS-staining buffer (PBS containing 0.01% sodium azide and 5% BSA with 0.2% saponin) for 15 min at RT. Detection of infected cells was performed by incubation with an anti-p72 monoclonal antibody (17LD3, gifted by Ingenasa, Madrid, Spain) diluted 1:100 in PBS-staining buffer for 30 min at 4 °C, followed by incubation with an anti-mouse Alexa Fluor-488 diluted 1:500 in PBS-Staining buffer using the same conditions. Finally, the gating was set by the uninfected control and 20,000 events/cells per sample were analyzed in a FACS Calibur flow cytometer (BD Science, San Jose, CA, USA) to determine the percentage of ASFV-infected cells. All FACS analyses were displayed as the average percentage of p72 expression representing infected cells. As a positive control, serum from a pig infected with ASFV and its respective corresponding pre-immune sera, as negative control, were used. The assay was performed as biological triplicates and duplicates, for Groups 1 and 2 sera, respectively.

### 2.8. Quantitative Real-Time PCR Assay for ASFV DNA

The detection and quantification of ASFV DNA for blood samples from challenged pigs was performed using a quantitative real-time PCR assay. DNA was purified by automated magnetic bead extraction on the KingFisher Duo Prime Purification System using the DNeasy Blood and Tissue Kit with the MagAttract Suspension G (Qiagen). The extraction was performed according to manufacturer’s instruction with minor modification. Briefly, 200 µL of EDTA blood sample was added to 200 µL of AL buffer supplied by the kit for cell lysis, and heat treated at 70 °C. Subsequently, the lysed sample was added to 100 µL AL buffer with 50 µL of magnetic beads. Two-hundred microliters (200 µL) of molecular grade isopropanol (ThermoFisher Scientific, Waltham, MA, USA) was added prior to the automated magnetic bead extraction. DNA bound to beads was washed two times with the kit supplied AW1 buffer (750 µL), once with the kit supplied AW2 buffer (750 µL), and followed by a final wash with 200 proof molecular grade ethanol (750 µL). Following a five minute drying time, DNA was eluted in 100 µL of elution buffer. Negative (molecular grade water) and positive (ASFV positive sample) controls were included with each extraction.

The sequences of primers and the probe for the detection of ASFV p72 gene were described previously [43]. Real-time quantitative PCR (qPCR) was conducted using PerfeCTa^®^ FastMix^®^ II (Quanta Biosciences, Gaithersburg, MD, USA) using a CFX96 Touch™ Real-Time PCR Detection System (Bio-Rad, Hercules, CA, USA). Quantitative qPCR was performed using 2.5 µL of DNA template, 20 pmol each of primers (Integrated DNA Technology, Coralville, IA, USA), and 7 pmol of probe (Thermo Fisher Scientific) in a final reaction volume of 20 µL. Each reaction was performed in triplicate. Thermocycling parameters were 95 °C for 5 min, followed by 45 cycles of 95 °C for 10 s and 60 °C for 1 min. Negative and positive controls were included in each PCR run and consisted of molecular grade water and ASFV positive amplification control, respectively. The Ct 35 was selected as the Ct cutoff value based on the analytical limits of the qPCR assay. ASFV copy number (CN) was calculated via a standard curve generated using ten-fold serial dilutions of the quantitated ASFV DNA positive control.

### 2.9. Statistical Analysis

The data was analyzed using GraphPad Prism 6 Software (GraphPad Software, San Diego, CA, USA), by the respective statistical tests and methods indicated in the figure legends. Results are shown as group means with standard deviation. 

## 3. Results

### 3.1. Evaluation of Humoral Immune Responses after Vaccination With ASFV-Recombinant Proteins and pcDNAs 

**Figure 1:**
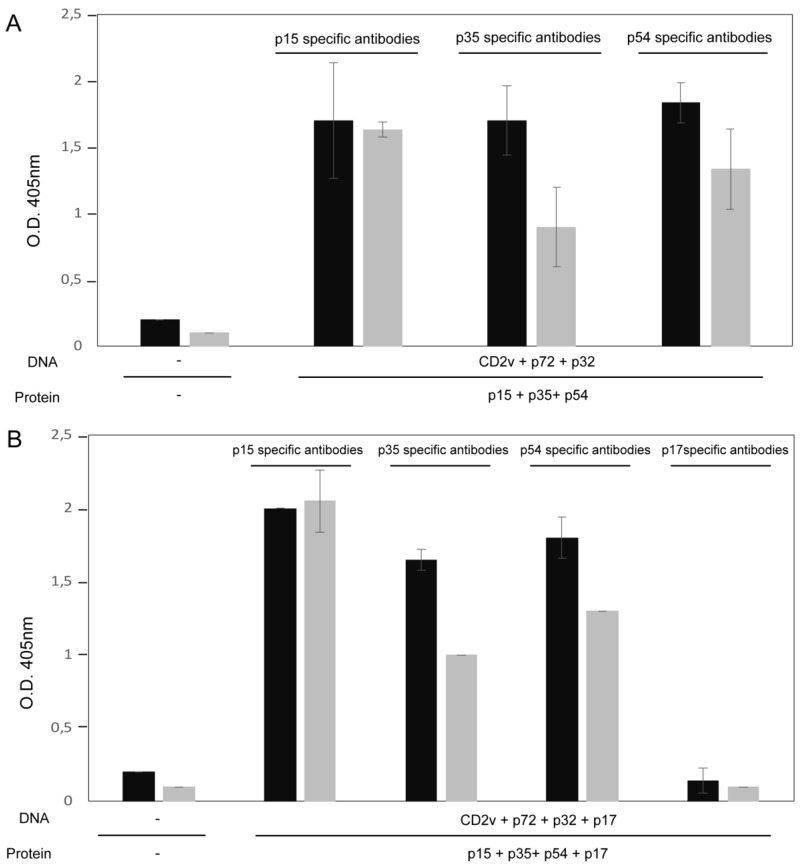
ASFV-specific antibodies in sera of immunized pigs detected by recombinant viral protein ELISAs. Sera from pigs immunized 3 times with the specified recombinant viral proteins or pcDNAs, and non-immunized pigs were tested for p15, p35, p54, or p17 specific antibodies by single antigen ELISAs. Sera collected at 49 days after initial vaccination were tested at 1/20 (black bars) or 1/200 (grey bars) dilutions. Antibody levels are represented by mean optical density with standard deviation. Immunized pigs from groups 1 and 2 are shown in panels A and B, respectively. Mean reactivity of non-vaccinated pig sera against each of the recombinant proteins is represented by the first set of bars in each graph. The results represent biological triplicates and duplicates, for sera from Groups 1 (A) and 2 (B), respectively.

A mixture of ASFV antigens consisting of recombinant proteins (p15 + p35 + p54, +/− p17) with pcDNAs (CD2v + p72 + p32, +/− p17) were used to vaccinate two groups of pigs as indicated in Table 2. Recombinant viral protein based ELISAs were developed to assess antibody induction by the combined protein-DNA vaccination approach. Sera from pigs immunized three times and non-vaccinated controls were tested by ELISA against individual p15, p35, p54, and p17 recombinant proteins. The results showed similar antibody responses in both groups of immunized pigs against ASFV proteins p15, p35, and p54 at 49 days post initial vaccination (Figure 1). Reactivity appeared to be highest against p15 with saturation observed at 1/20 and 1/200 sera dilutions, followed by p54, and p35. Reactivity against the p17 structural protein by ELISA matched background levels, indicating that p17 is either a poor antigen or possible interference by other antigens exists. However, p17 recombinant protein was weakly recognized by Western blot with immunized pig serum (Supplementary Figure S1). It should be noted that the Western blots were developed under denaturing conditions; thus, the reactive antibodies found by this technique most likely recognize linear, but not conformational epitopes. 

### 3.2. IFN-γ Secreting Cells (ELISPOT Assay)

**Figure 2:**
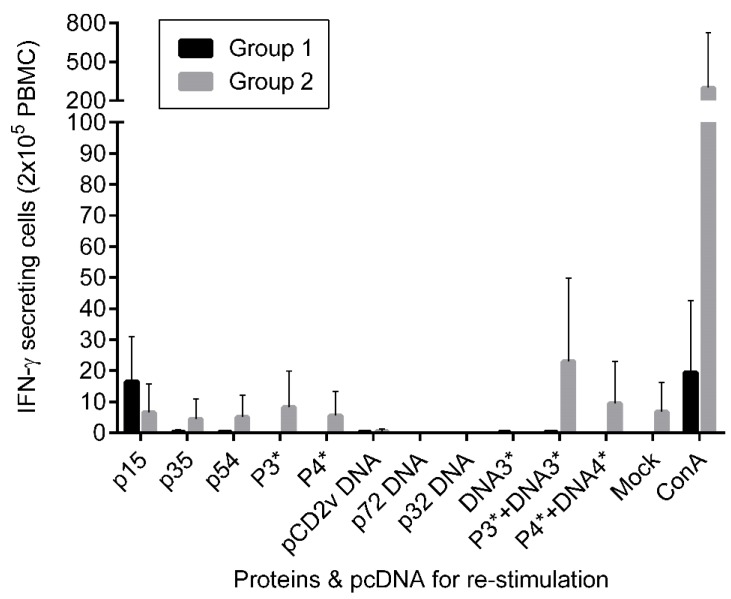
IFN-γ secreting cells from PBMCs after re-stimulation with antigens determined by ELISPOT assay. PBMCs derived from Group1 (n=3) and Group2 (n=2) immunized piglets two weeks after the final boost were re-stimulated with the respective antigens and pcDNAs. P3* = P15+P35+P54 proteins; P4* = p15 + p35 + p54 + p17 proteins; DNA3* = CD2v + p72 + p32 pcDNAs; DNA4* = CD2v + p72 + p32 + p17 pcDNAs; ConA = concanavalin A (20ug) was used as a positive control. The pcDNAs and media only mock were included as negative controls. Mean and standard deviations are shown. No significant differences were found. The results represent biological triplicates and duplicates, for PBMCs from Groups 1 (**A**) and 2 (**B**), respectively.

IFN-γ secreting cells in PBMCs from immunized pigs were analyzed by ELISPOT assay. PBMCs were isolated from blood of immunized pigs at 49 day post vaccination (dpv), two weeks after the final boost, before the animals were moved to the BSL-3 facility. This time point allowed for both immune development and the ability to analyze samples outside of high containment. The isolated PBMCs were subsequently stimulated with the same antigens as used for immunization. As shown in Figure 2, only low numbers of IFN-γ secreting cells specific for ASFV antigens, and none or negligible numbers specific for the pcDNAs were observed. In Group 1, 2/3 of the immunized pigs produced IFN-γ secreting cells specific for the p15 protein. In Group 2, antigen-specific IFN-γ secreting cells were detected from only one pig. Only the combinations of the proteins p15 + p35 + p54, and proteins/pcDNAs combinations p15 + p35 + p54/CD2v + p72 + p32, and p15 + p35 + p54 + p17/CD2v + p72 + p32 + p17 were found at numbers above the mock control. 

### 3.3. Sera from Immunized Pigs Enhances ASFV Infection in vitro (Neutralization Assay)

**Figure 3:**
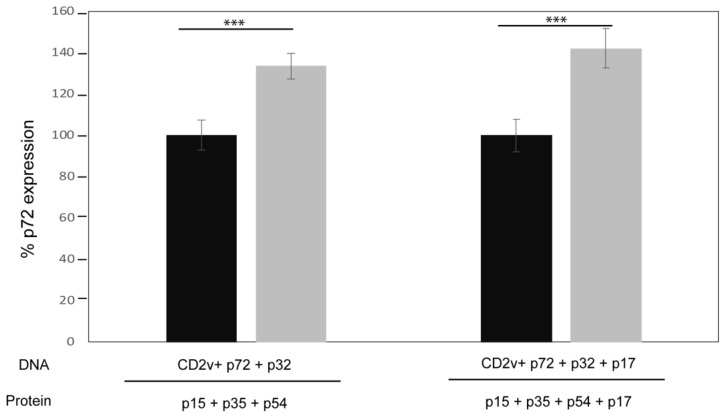
Sera from immunized pigs enhances ASFV infection in vitro. ASFV strain Ba71V pre-incubated with either pre-immune sera (black bars) as a control, or immune sera (grey bars) collected at 49 days post vaccination was used to infect Vero cells. After 16 hours post infection the percentage of p72 expression was determined by FACS using a specific monoclonal antibody (17LD3) to measure virus infection. Mean with standard deviations are shown for each vaccinated group of pigs. Significance was determined by student t test (***; *p*-value < 0.001). The results represent biological triplicates and duplicates, for Groups 1 (left) and 2 (right), respectively.

The presence of ASFV neutralizing antibodies in the sera of immunized pigs was determined by incubating cells with pre-incubated sera and virus combinations, followed by FACS analysis of cells for the expression of the p72 viral capsid protein as a measure of infection. To demonstrate the capacity of this assay to determine virus neutralization, hyper-immune serum from an ASFV-infected pig resulted in around 80% reduction of ASFV infection in Vero cells compared to the pre-immune serum [34]. Using this method, sera collected from pigs before vaccination (pre-immune sera) as a negative control and post-vaccination sera following the third booster immunization (immune sera, before challenge) were tested for neutralizing antibodies. Interestingly, the immune sera from the vaccinated pigs resulted in a 30–40% increase of cells expressing p72 compared to the pre-immune sera, for both vaccination groups (Figure 3). These results suggest that the post-vaccination sera enhanced virus infection or virus replication, at least in vitro.

### 3.4. Clinical Observation after Challenge with Virulent Arm07 ASFV Strain

**Figure 4:**
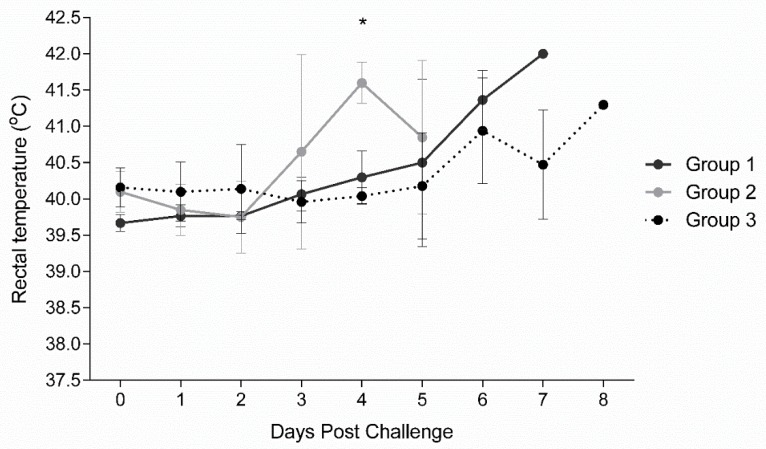
Rectal temperature responses in pigs challenged with ASFV Armenia 2007. Mean rectal temperature responses with standard deviation (SD) in vaccinated (Group 1 and Group 2) and non-vaccinated pigs (Group 3) are shown. Statistical significance was determined by multiple comparison *t* test using the Holm-Sidak method, with alpha = 5.000%, and each row analyzed individually, without assuming a consistent SD. Significance (*; *p*-value 0.00009) was found at day 4 between Groups 2 and 3.

**Figure 5:**
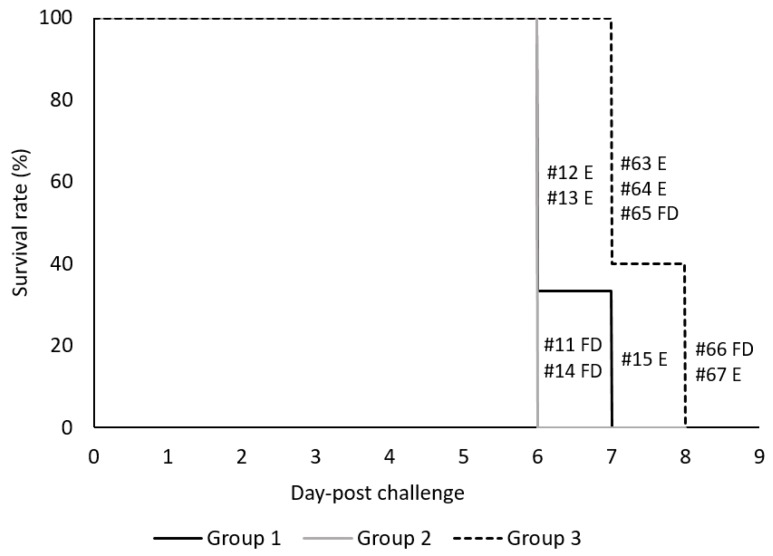
Survival rate (%) of vaccinated and control pigs following challenge with ASFV Armenia 2007. The black line denotes vaccinated Group 1 (#12, 13, 15), the grey line vaccinated Group 2 (#11, 14) and the dashed line represents the non-vaccinated control Group 3 (#63–67). Individual pig number defines whether the animal was found dead (FD) or euthanized (E). Survival curves between Groups 2 & 3 were found statistically significant (*p*-value of 0.0143) by the Gehan-Breslow-Wilcoxon test using multiple comparison and the Bonferroni-corrected threshold of 0.0167. The *p*-values for comparisons between Groups 1 & 3 and 1 & 2 were 0.0388 and 0.4142, respectively.

Following three immunizations at days 0, 21, and 35, all vaccinated and non-vaccinated control pigs were challenged via IM with high dose (360 HAU) of virulent ASFV Arm07 virus at 3 weeks after the last immunization (0 dpc). Rectal temperatures of individual pigs were measured daily and twice per day after 5 dpc. All ASFV inoculated animals developed fever (temperatures higher than 40 °C) during the observation period of up to eight days (Figure 4). Following challenge, the Groups 1 and 2 vaccinated animals were observed with higher temperatures than the non-vaccinated Group 3 animals starting at 3 dpc. The mean temperatures of Group 2 pigs peaked at 4 dpc, three to four days earlier than Group 1 or 3, respectively. All challenged pigs, independent of their vaccination status, developed ASFV-specific clinical signs which were scored as described in the Supplementary Table S1 and are summarized in Table 3. Clinical scores were observed for all Group 1 and 2 vaccinated pigs by 4 dpc, and all Group 3 non-vaccinated pigs by 6 dpc. Group 2 pigs had higher mean clinical scores than either Group 1 or 3 from 3 to 5 dpc. Group 1 mean clinical scores were higher than Group 3 on both 6 and 7 dpc. Clinical signs observed consisted of lethargy and reduced liveliness (presented in all the animals), breathing difficulties and coughing (2/5 vaccinated and 2/5 non-vaccinated pigs), ocular/nasal discharge (0/5 vaccinated and 4/5 non-vaccinated pigs), reduction on the body shape (3/5 for vaccinated and 3/5 for non-vaccinated pigs) and skin lesions with cyanosis of the skin (4/5 vaccinated and 5/5 non-vaccinated pigs). Neurological signs or digestive problems were not observed. The detailed survival time for each animal is presented in Figure 5. Specifically, Group 1 vaccinated pigs were euthanized at 6 or 7 dpc, Group 2 pigs were found dead at 6 dpc, whereas Group 3 non-vaccinated control pigs were found dead or euthanized on 7 and 8 dpc. These results show that vaccinated animals were not protected against high dose virulent ASFV challenge. Moreover, vaccination appeared to result in more rapid morbidity and mortality following virulent ASFV challenge.

### 3.5. Micro- and Macroscopic Pathology after Challenge 

Pigs found dead or those euthanized due to ASF were necropsied to assess the macroscopic and microscopic pathology as described in Supplementary Table S2. Pigs from each group demonstrated mild to moderate gross lesions such as cutaneous hyperemia, edema, and hemorrhages in lymph nodes and tonsils, splenomegaly, hepatopathy, and pulmonary edema, consistent with acute ASFV infection. All Groups 1 and 2 vaccinated pigs showed moderate overall lesion scores (5/5), while 2/5 Group 3 non-vaccinated pigs had mild overall lesion scores and 3 with moderate lesions scores. Although overall gross pathology scores among groups were not statistically significant, lesion severity tended to be more severe in the vaccinated groups. Overall mean gross scores for Group 1 was 29.7 ± 9.87 (mean ± standard deviation), 31.5 ± 7.78 for Group 2, and 18.6 ± 13.94 for Group 3 (Table 3). Gross lesions include cutaneous hyperemia with occasional petechial and ecchymotic hemorrhages, coagulopathy (measured by moderate to severe hemorrhage at venipuncture sites and within body cavities), moderate to severe edema and patchy to diffuse hemorrhage within one or more lymph nodes and tonsils. Visceral lesions included marked splenic congestion and necrosis, and moderate to severe pulmonary edema occasionally occupied by patchy pulmonary hemorrhage and lobar consolidation due to interstitial pneumonia. Some pigs displayed hepatopathy with hemorrhage and edema of the biliary tree and moderate to several renal congestion. Mild lesions were characterized as mild edema and patchy hemorrhage of the lymph nodes and mild pulmonary edema. One control pig #64, had mild lesions more consistent with lymphoid proliferation rather than hemorrhage and necrosis; with moderately enlarged lymph nodes containing multiple proliferative nodules and a large firm spleen with prominent lymphoid tissue. 

Histological analysis was performed on liver, lung, kidney, spleen, and tonsil, as well as mandibular, renal, gastrohepatic, prescapular, and mesenteric lymph nodes. Scores of vaccinated pigs in Group 1 consisted of two animals with moderate and one animal with moderate to severe scores, and Group 2 had one moderate and one with severe histopathological lesions. The Group 3 non-vaccinated animals had one animal with mild, two with moderate, one with moderate to severe and one with severe lesions. Although overall histopathology scores among groups were not statistically significant, lesion severity tended to be more severe in the vaccinated group. Overall mean histological score for Group 1 was 62.2 ± 10.77 (mean ± standard deviation), 66 ± 9.90 for Group 2, and 57.9 ± 19.32 for Group 3 (Table 3). However, mean pulmonary lesion scores were significantly worse in the non-vaccinated control group (*p* < 0.001) which is attributed to severe pulmonary lesions in two pigs within this group (#65 and #66). In summary, vaccinated animals showed more severe gross and histopathological lesions when compared to non-vaccinated animals (Table 3), indicating that immunopathological events might have contributed to the pathogenesis of ASFV infection in vaccinated pigs.

### 3.6. Presence of ASFV DNA in the Blood of Infected Animals 

**Figure 6:**
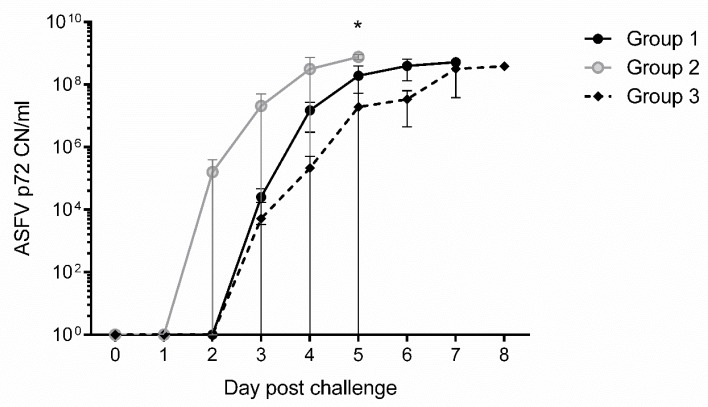
ASFV p72 copy number (CN/mL) in whole blood of swine following challenge with virulent ASFV Armenia 2007. The mean quantitative qPCR determined ASFV p72 CNs are shown with standard deviation (SD) for Groups 1 and 2 vaccinated animals, and Group 3 challenge controls. Each reaction was performed in triplicate. Statistical significance was determined by multiple comparison *t* test using the Holm-Sidak method, with alpha = 5.000%, and each row analyzed individually, without assuming a consistent SD. Significance (*; *p*-value 0.00005) was found at day 5 between Groups 2 and 3.

To determine viremia status, ASFV DNA was quantitated from whole blood using a quantitative real-time qPCR assay targeting the ASFV p72 gene. The analytical limits of detection with this assay for Arm07 is approximately 10 copies per reaction. All animals, except one non-vaccinated control pig (#64), became ASFV-positive in whole blood by real time qPCR. Temporally, on average, whole blood samples were positive at 2–3 dpc for Group 2 and 3–4 dpc for Group 1 vaccinated pigs, and at 3–5 dpc for Group 3 non-vaccinated, challenge control pigs. The ASFV copy number increased exponentially on 3-5 dpc for vaccinated animals and on 3–7 dpc for challenge control pigs as shown in Figure 6. Blood of non-vaccinated control pig #64 remained negative for ASFV DNA by real-time qPCR. However, low copy numbers (20–200) of ASF p72 was detected in tissues tested including lymph nodes, tonsils, spleen, heart and liver, indicating low level ASFV infection in this animal. These results suggest that vaccinated pigs overall had an earlier onset of viremia and have higher viral loads at time of death or euthanasia, as shown by the higher ASFV DNA copy number in their blood, than non-vaccinated control pigs following challenge with Arm07 (Figure 6).

## 4. Discussion

In the current pilot study, pigs were immunized with a cocktail of ASFV pcDNAs and recombinant proteins. The antigens selected included those previously investigated (p32, p54, p72, CD2v) by others, as well as, viral structural proteins (p15, p17, p35) not before incorporated in ASFV vaccine formulation. The aim of the pcDNA constructs was to prime the CTL response. While the antigens in this study were based on ASFV genotypes I and II strains, the percent sequence identity of these genes are highly conserved, with the exception of CD2v (Table 1). The pcDNA encoding CD2v was included to enhance the immune response when combined with the other ASFV antigens, which has been shown previously by Argilaguet et al., 2012 [25]. Furthermore, using the same experimental design and methods, we previously found that sera against p35 recombinant protein combined with pcDNA-CD2v resulted in increased neutralizing activity [34]. Likewise, pcDNA-p32 combined with p15 recombinant protein also appeared to increase virus neutralizing activity [34]. The addition of p17, was also hypothesized to induce neutralizing antibodies [32]. Yet, stimulation of CTLs was minimal based on the few antigen-specific INF-γ secreting cells detected following three immunizations with the vaccine cocktails (Figure 2), and sera from both groups of immunized pigs were unable to neutralize virus infection (Figure 3). All immunized pigs did develop antigen-specific antibodies against the recombinant proteins p15, p35 and p54. However, no specific antibody response against p17 was detected by ELISA (Figure 1), and only weak antibody response to the recombinant protein was detected in sera from an immunized pig (Supplementary Figure S1). 

Vaccine efficacy was tested by challenge with virulent ASFV Arm07 strain. Following challenge, immunized pigs were not protected from infection. In comparison to the non-immunized controls, vaccinated animals displayed more rapid onset of viremia (Figure 6), enhanced clinical disease (Figure 4 and Table 3) and more rapid death (Figure 5). ASFV induced pathology was also more apparent in the vaccinated pigs. While Group 2 pigs did exhibit earlier onset of ASFV infection, disease and death compared to Group 1, it is difficult to say whether p17 played a critical role given the low sampling number in each group, which is acknowledged as a limitation of this work. Since this was a pilot study and the significant cost associated with BSL3 work, animal numbers were restricted for this study. Overall, the results showed that even though antigen-specific antibodies were detected in the vaccinated pigs, they lacked the ability to neutralize virus in vivo and in vitro, and appeared to enhance ASFV infection. The results emphasize the need to improve antigen selection, and to further investigate the mechanism of the ASFV-specific immune response in ASFV pathogenesis. 

Relatively few reports have described the mechanism of ASFV neutralization, and the most accepted hypothesis is that ASFV induces antibodies that are not fully neutralizing in vitro or protective in vivo [17,42,43,44,45]. However, several laboratories have demonstrated that ASFV can be neutralized by both, monoclonal antibodies and immune sera from convalescent swine infected with homologous or heterologous ASFV strains [14,15,18,46,47]. Importantly, a fraction of virus (about 10–20%) which cannot be neutralized by antibodies has also been reported in these studies. Interestingly, our neutralization assay results for sera from pigs vaccinated with our cocktail of ASFV antigens actually showed enhanced virus infection in vitro (Figure 3).

Furthermore, accelerated infection in vivo observed in vaccinated pigs was evidenced by earlier onset of clinical signs, viremia and death compared to the non-vaccinated, challenge controls. An earlier onset of disease in immunized animals following virulent challenge has been previously observed for ASFV infection during an inactivated vaccine trial [48]. As suggested previously by Blome and colleagues, we also hypothesize that this might be due to antibody-dependent enhancement (ADE) of infection. It is known that cell entry of microorganisms that replicate in macrophages like ASFV, can be mediated via IgG antibody complexes through Fcγ-receptor signaling and lead to increased production of pathogens by the infected cells [49,50]. The early onset of viremia in vaccinated animals as observed here could be due to ADE of ASFV infection in target macrophages. Similar events are described for other virus infections like Dengue, feline infectious peritonitis virus, HIV, and others [50]. 

Interestingly, we observed increased infection in vitro using Vero cells which lack Fcγ-receptors. Enhancement of virus infection in the presence of immune sera has also been reported for Vero and other Fcγ-negative cells infected with Dengue [51] and West Nile virus [52]. Recently, an Fcγ-independent mechanism of ADE for flavivirus infection was reported, in which antibody-induced conformational changes of virus envelope led to increased virus binding to cells [53]. The results from this study and the previous report by Blome and colleagues [48] presents an interesting aspect of ASF pathogenesis that has not yet been thoroughly investigated. Additional studies are necessary to elucidate the mechanism of enhancement of ASFV infectivity in vitro and ASFV virulence in vivo.

## 5. Conclusions

More knowledge is needed in order to define the individual contributions of the immune mechanisms and target antigens involved in protection against ASF. The generation of a successful subunit vaccine will depend on the identification of viral targets which, together with an optimal delivery method and adjuvant, allow the correct presentation of the viral antigens and the activation of protective immune responses. The fact that vaccination accelerates ASF disease in this study, suggests that immunopathological mechanisms, including ADE of infection in vitro and possibly in vivo, might be part of ASF pathogenesis. Additional studies to elucidate the underlying mechanism are critically needed in order to develop a rationally designed efficacious and safe ASFV vaccine.

## Figures and Tables

**Table 1:** Percent identities between strains of ASFV antigens used in this study.

ASFV Gene	Encoded Protein	ASFV Strain	Genotype	Expression	% Identity of Ba71V to Georgia/2007	
					Nucleotide	Amino Acid
CP530R	p15	Georgia/2007	II	Baculovirus	98	97*
CP530R	p35	Georgia/2007	II	Baculovirus	98	97*
E183L	p54	Georgia/2007	II	Baculovirus	96	96
D117L	p17	Ba71V	I	pcDNA3.1	97	10
CP204L	p32	Ba71V	I	pcDNA3.1	98	98
B646L	p72	E70	I	pcDNA3.1	99**	na**
EP402R	CD2v	Ba71V	I	pcDNA3.1	81	57

* = based on pp62; ** = % identity of E70 to Georgia/2007; na = not available.

**Table 2:** Experimental groups for evaluation of immunogenicity and protective effect of vaccination with combinations of ASFV proteins and DNA plasmid constructs.

Animal Groups	Antigens for Vaccination on Days 0, 21, and 35		Pig Identification Numbers
	Recombinant Protein	pcDNA Construct	
Group 1	p15 + p35 + p54	CD2v + p72 + p32	12, 13, 15
Group 2	p15 + p35 + p54 + p17	CD2v + p72 + p32 + p17	11, 14
Group 3	Non-vaccinated controls		63, 64, 65, 66, 67

**Table 3:** Mean clinical and pathological scores post challenge with ASFV Armenia 2007.

Scores	dpc	Group 1	Group 2	Group 3
Observed clinical score	0	0.0 ± 0.00	0.5 ± 0.71	0.8 ± 0.45
	1	0.0 ± 0.00	1.0 ± 1.41	0.8 ± 1.10
	2	0.0 ± 0.00	0.5 ± 0.71	1.0 ± 1.22
	3	0.7 ± 0.58	2.0 ± 2.83	1.0 ± 1.00
	4	1.7 ± 0.58	4.5 ± 0.71*	1.2 ± 0.84*
	5	2.67 ± 2.08	9.5 ± 0.71*	3.4 ± 1.95*
	6	10.33 ± 3.51	na	6.4 ± 3.58
	7	25.67 ± 10.97	na	15.2 ± 9.52
	8	na	na	28.8 ± 7.16
Gross pathological score	5–8	29.7 ± 9.87	31.5 ± 7.78	18.6 ± 13.94
Histopathological score	5–8	62.2 ± 10.77	66 ± 9.90	57.9 ± 19.32

Mean ± standard deviation (SD); dpc = days post challenge; na = no surviving animals. Clinical and pathological scoring details are provided in supplementary Tables S1 and S2. Significance found between Groups 2 and 3 clinical scores at days 4 and 5 (*; *p*-values of 0.004 and 0.009, respectively). Statistical significance was determined by multiple comparison *t* test using the Holm-Sidak method, with alpha = 5.000%, and each row analyzed individually, without assuming a consistent SD.

## Supplementary Materials

The following are available online at http://www.mdpi.com/2076-393X/7/1/12/s1, Figure S1. Recombinant viral protein p17 expression and recognition by immunized pig serum, Table S1. Clinical signs and scoring parameters, Table S2. Evaluation criteria for gross and histological pathology and score.
