# Supplementary material for: DNA-Protein Vaccination Strategy Does Not Protect from Challenge with African Swine Fever Virus Armenia 2007 Strain
Source: Vaccines (Basel). 2019 Jan 28;7(1):12. doi: 10.3390/vaccines7010012 (PMC6466342; doi:10.3390/vaccines7010012)
Supplement: Supplementary file 1 [file vaccines-07-00012-s001.pdf]

vaccines

Article

DNA-Protein Vaccination Strategy Does Not Protect from Challenge with African Swine Fever Virus Armenia 2007 Strain

Sun-Young Sunwoo [1,†], Daniel Pérez-Núñez [2,†], Igor Morozov [1], Elena G. Sánchez [2], Natasha N. Gaudreault [1], Jessie D. Trujillo [1], Lina Mur [1], Marisa Nogal [2], Daniel Madden [1], Kinga Urbaniak [1], In Joong Kim [1], Wenjun Ma [1], Yolanda Revilla [2,*], Juergen A. Richt [1,*]

1 Department of Diagnostic Medicine & Pathobiology, College of Veterinary Medicine, Kansas State University, K2224 Mosier Hall, 1800 Denison Ave, Manhattan, KS 66506, USA; sunwoosy@gmail.com (S.-Y.S); imorozov@vet.k-state.edu (I.M.); nng5757@vet.k-state.edu (N.N.G.); jdtrujillo@vet.k-state.edu (J.D.T.); linamurvet@gmail.com (L.M.); dwmadden@vet.k-state.edu (D.M.); kinia-u@wp.pl (K.U.); lui0125@gmail.com (I.J.K.); wma@vet.k-state.edu (W.M.)

2 CBMSO-CSIC-UAM, C/Nicolás Cabrera 1, Campus de Cantoblanco, Madrid 28049, Spain; daniel_perez@cbm.csic.es (D.P.-N.); elena_garcia@cbm.csic.es (E.G.S.); marisa_nogal@cbm.csic.es (M.N.)

* Correspondence: jricht@vet.k-state.edu (J.A.R.); yrevilla@cbm.csic.es (Y.R.); Tel.: +1-785-532-2793 (J.A.R.); +34-91-196-4570 (Y.R.)

† These authors contribute equally to this paper.

Supplementary Materials

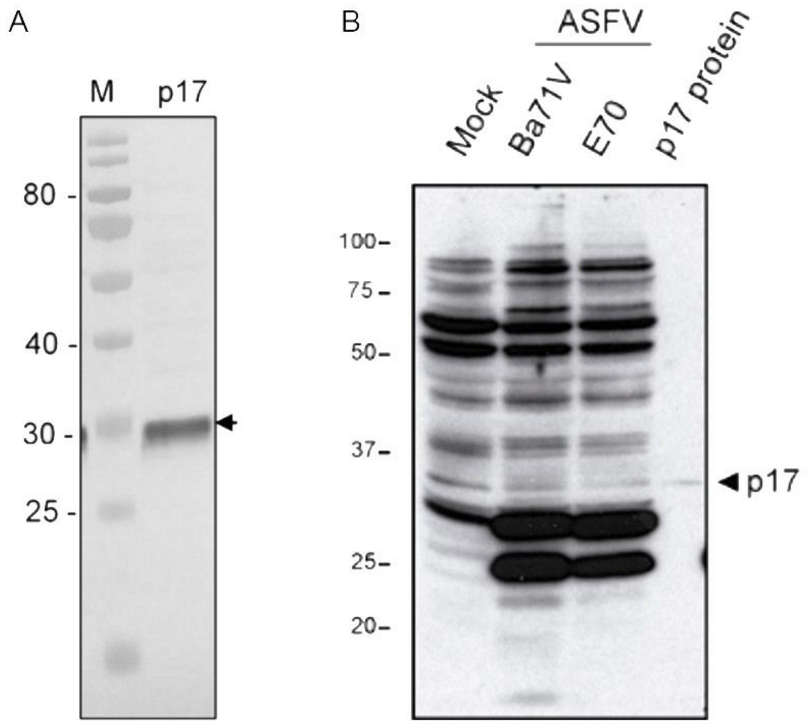

Figure 1. Western Blot of Recombinant viral protein p17 expression and recognition by hyperimmune swine serum. (A) Expression of recombinant p17 protein immunogen evaluated by western blot with anti-GST tag antibodies. (B) Expression of viral p17 protein from Vero or COS-7 cells infected with the respective Ba71V or E70 ASFV strains evaluated by western blot with serum from a pig in Group 2 immunized with the cocktail that included p17. Recombinant protein used to vaccinate swine in this study is in the last lane. M = protein ladder; kDa represented on the left.

vaccines MDPI

Table 1. Clinical signs and scoring parameters.

Clinical Signs	Severity (scoring)				
	0	1	2	3	4
Liveliness	No Abnormality	Reduced liveliness, stands w/o help	Sluggish, stands with help	Dormant, refusal to stand with or w/o help	Not used
Body Shape	No Abnormality	Empty Stomach (sunken flanks)	Empty Stomach with weight loss indicators	Wasting (ribs, vertebrae visible, rough hair)	Not used
Breathing	No Abnormality	Increased Respiratory Rate	Significantly increased respiratory rate, abdominal breathing	Difficult Breathing (open mouth breathing, wheezing coughing)	Not used
Neurological Signs	No Abnormality	Stumbling gait, quickly corrected	Ataxia/paresis of the hindquarters, continues to walk	Paralysis of the hindquarters, unable to stand, convulsions	Not used
Skin	No Abnormality	Red skin, cyanosis of the skin (<10%)	Cyanosis of the skin(10-25%), occasional skin bleeding	Cyanosis of the skin (>25%), large blue/black spots, large subcutaneous bleeding, necrosis/ulceration	Not used
Digestive Symptoms	No Abnormality	Diarrhea (<24 Hours)	Diarrhea(>24hours), Occasional vomiting	Bloody diarrhea, frequent vomiting	Not used
Ocular/Nasal Discharge	No Abnormality	Thin discharge from nose and/or eyes (w/o admixtures)	Thick discharge from nose and/or eyes (no blood)	Bloody discharge from nose and/or eyes	Not used
Fever	Temp of 38–39 °C	Temp of ≥ to 40 °C	Temp of ≥ to 40 °C for two sequential days	Temp of ≥ to 41 °C	Temp of <38 °C

Table 2. Evaluation criteria for gross and histological pathology and score. Scoring was modified from Galindo-Cardiel et al. 2013.

	Evaluation criteria and score	
	**Gross pathology**	**Histological pathology**
**Body condition**	Normal (0); Mild loss (1) vertebrae and pelvic bones detectable by firm palpation and ribs not overtly visible but palpable; Moderate loss (2) vertebrae and pelvic bones visible to prominent ribs visible; Severe loss of body condition (3) vertebrae and pelvic bone obvious with muscle wasting, prominent ribs.	
**Integument**	Normal (0), pronounced hyperemia (1), petechia/ecchymosis (2), cyanosis/infarction (3).	
**Cardiovascular**	Subcutaneous/intramuscular/abdominal/articular central nervous system: hemorrhage and edema mild (1), moderate (2), severe (3).	

**Liver**	Hepatopathy (enhanced lobular patter, intrahepatic mottling, congestion, texture (friability) and Bile duct and vascular hilus, edema and/or hemorrhage with prominent vasculature.	1) vascular/biliary changes: portal angiectasia and sinusoidal dilation, fibrin thrombi, bile duct hypertrophy/hyperplasia, 2) hepatopathy: portal and sinusoidal infiltrates or inflammatory cells, apoptosis and/ or necrosis of hepatocytes, kupffer cell hypertrophy, biliary epithelium, or portal vasculature.
**Lung**	1) Pulmonary collapse or lack of it leading to rib impression formation, 2) edema, 3) congestion and or hemorrhage, 4) pneumonia and 5) the formation of pleural effusion or 6) hemorrhage.	1) Interstitial and alveolar edema (less than 15% of sections affected are classified as mild, 15-30% as moderate and >30% as severe), 2) congestion with angiectasia, vasculopathy and hemorrhage (moderate and severe), 3) pneumonia characterized multifocal to coalescing inflammatory infiltrates within alveolar spaces and wall, peribronchiolar and perivascular.
**Spleen**	1) Congestion and/or hemorrhage and 2) texture/necrosis (spongey and friable) or infarction.	Ratio of white pulp to red pulp (<1:4 as mild, 1:5 to 1:8 as moderate and >1:9 as severe. This includes when present represents an estimate of lymphoid depletion and hypertrophy of the reticulo-endothelial system, , 2) necrosis/ infarcts, 3) Lymphocytolysis: individual lymphoid or histiocytic cell necrosis/apoptosis, 4) vascular damage: angiectasia, red pulp congestion, hemorrhage, fibrinoid necrosis of vascular walls/ inflammatory infiltrates.
**Lymph nodes/tonsil**	1) Edema,2) congestion, 3 hemorrhage, 3) necrosis, 4) enlargement- swelling or nodular proliferation,	
**Kidney**	Congestion, hemorrhage and edema; multifocal, cortical, medullary, and/or subcapsular.	Congestion, hemorrhage and edema within the medulla or cortex and interstitial inflammation and necrosis of the tubular epithelium with cast formation.
